# Evaluation of Recombinant Botulinum Neurotoxin Type A1 Efficacy in Peripheral Inflammatory Pain in Mice

**DOI:** 10.3389/fnmol.2022.909835

**Published:** 2022-05-26

**Authors:** Beatrice Oehler, Cindy Périer, Vincent Martin, Amy Fisher, Stéphane Lezmi, Mikhail Kalinichev, Stephen B. McMahon

**Affiliations:** ^1^Wolfson Center of Age-Related Diseases, IoPPN, Health and Life Science, King’s College London, London, United Kingdom; ^2^Department of Anaesthesiology, Heidelberg University Hospital, Heidelberg, Germany; ^3^Ipsen Innovation, Les Ulis, France; ^4^Transpharmation Ltd., London, United Kingdom

**Keywords:** inflammation, nociception, analgesia, GCaMP, *in vivo* calcium imaging, hyperalgesia, sensitization, cleaved SNAP-25

## Abstract

Well-established efficacy of botulinum neurotoxin type A (BoNT/A) in aesthetic dermatology and neuromuscular hyperactivity disorders relies on canonical interruption of acetylcholine neurotransmission at the neuromuscular junction at the site of the injection. The mechanisms and the site of activity of BoNT/A in pain, on the other hand, remain elusive. Here, we explored analgesic activity of recombinant BoNT/A1 (rBoNT/A1; IPN10260) in a mouse model of inflammatory pain to investigate the potential role of peripheral sensory afferents in this activity. After confirming analgesic efficacy of rBoNT/A1 on CFA-induced mechanical hypersensitivity in C57Bl6J mice, we used GCaMP6s to perform *in vivo* calcium imaging in the ipsilateral dorsal root ganglion (DRG) neurons in rBoNT/A1 vs. vehicle-treated mice at baseline and following administration of a range of mechanical and thermal stimuli. Additionally, immunohisochemical studies were performed to detect cleaved SNAP25 in the skin, DRGs and the spinal cord. Injection of CFA resulted in reduced mechanical sensitivity threshold and increased calcium fluctuations in the DRG neurons. While rBoNT/A1 reduced mechanical hypersensitivity, calcium fluctuations in the DRG of rBoNT/A1- and vehicle-treated animals were similar. Cleaved SNAP25 was largely absent in the skin and the DRG but present in the lumbar spinal cord of rBoNT/A1-treated animals. Taken together, rBoNT/A1 ameliorates mechanical hypersensitivity related to inflammation, while the signal transmission from the peripheral sensory afferents to the DRG remained unchanged. This strengthens the possibility that spinal, rather than peripheral, mechanisms play a role in the mediation of analgesic efficacy of BoNT/A in inflammatory pain.

## Introduction

Pain remains an area of considerable unmet medical need. The Global Burden of Disease survey, which has been running for several decades, continues to show that pain, and particularly musculoskeletal pain, is among the leading causes of disability worldwide, accounting for more impairment than cardiovascular diseases and Alzheimer’s disease combined (GBD 2019 Diseases and Injuries Collaborators, 2020). In developed countries, the anticipated prevalence of chronic pain is about 20% (Macfarlane, 2016; Kuehn, 2018). One major factor in poor pain management has been the slow development of novel classes of analgesic drugs. In this context there is a growing interest in analgesic potential of botulinum neurotoxin type A (BoNT/A), produced by *Clostridium botulinum*. For several decades this molecule has shown utility in aesthetic dermatology as well as in muscle or glandular hyperactivity conditions observed in patients with spasticity, dystonia, blepharospasm and others. It is well established that the activity of BoNT/A in these conditions relies on a blockade of acetylcholine neurotransmission at the neuromuscular junction in response to BoNT/A-mediated cleavage of the synaptic protein SNAP25.

There is growing clinical evidence that BoNT/A can show analgesic efficacy in a range of pain conditions which is largely independent of its muscle-relaxant properties (Finnerup et al., 2015; Herd et al., 2018). Based on several clinical trials and meta-analysis BoNT/A has been approved for treatment of some forms of migraine (Burstein et al., 2014; Blumenfeld et al., 2018; Herd et al., 2018). Randomized, placebo-controlled clinical trials have shown analgesic efficacy of BoNT/A in patients with posttraumatic, trigeminal and postherpetic neuralgias and diabetic neuropathy (Park and Park, 2017). Also, BoNT/A reduced pain in patients with low back pain, refractory shoulder pain as well as those suffering from osteoarthritic pain and pain linked to total knee arthroplasty (Singh et al., 2010; Intiso et al., 2015; Mendes et al., 2019). Analgesic efficacy of BoNT/A in the clinic is supported by the evidence from non-clinical studies in a wide range of rodent models of pain (Matak et al., 2019).

Despite growing evidence of analgesic efficacy of BoNT/A, the mechanisms and the site of activity remain elusive. On the one hand, there is evidence that analgesic efficacy of BoNT/A is linked to reduction of neurotransmitter release in peripheral terminals of nociceptors. BoNT/A blocked KCl-evoked release of CGRP and substance P in primary sensory neuronal cultures *in vitro* (Purkiss et al., 2000; Durham et al., 2004). Additionally, BoNT/A injection reduced bladder pain responses and inhibited CGRP release from nerve terminals (Chuang et al., 2004). BoNT/A injected *via* intraplantar route reduced nocifensive behaviors in the phase 2 of the formalin test and formalin-evoked glutamate release in the injected paw (Cui et al., 2004). On the other hand, there is evidence that analgesic efficacy of BoNT also involves spinal mechanisms. For example, intraplantar injection of BoNT/A reduced expression of Fos protein in the dorsal horn of the spinal cord and excitation of wide dynamic range neurons of the dorsal horn in phase 2 of the formalin response (Aoki, 2005). While initially spinal effects of BoNT were thought to be indirect, subsequent evidence of retrograde axonal transport of BoNT/A to the spinal cord, pointed at possibility of its direct central effects (Matak et al., 2014, 2019).

The aim of this study was to investigate whether analgesic efficacy of BoNT/A is associated with inhibition of functional hyperexcitability of peripheral nociceptors in a mouse model of inflammatory pain. We used intraplantar injection of complete Freund’s adjuvant (CFA) to induce mechanical allodynia detectable by the von Frey test and compared the efficacy of rBoNT/A1 to vehicle. Persistent excitability of many peripheral nociceptors to mechanical and thermal stimuli applied to the inflamed paw was monitored simultaneously by *in vivo* calcium imaging in the cell bodies located in the ipsilateral dorsal root ganglion (DRG). Responses of CFA-exposed animals treated with either rBoNT/A or vehicle were compared to those without inflammation. In addition, we evaluated the effect of rBoNT/A1 on CFA-induced inflammatory responses in the skin and assessed the presence of cleaved synaptosomal-associated protein 25 (c-SNAP25), the target of BoNT/A, in the injected hind paw, DRGs, and the spinal cord.

## Material and Methods

### Animals

Adult male and pregnant female C57/Bl6 mice (gestational days 14–16) were purchased from Charles River Laboratories (Harlow, UK). For behavioral experiments and histological analysis, male mice were group-housed at the animal facility of the Royal Veterinary College. Animals were acclimated for at least 7 days prior to the experiments. For *in vivo* imaging experiments, pregnant mice were single-housed at the animal facility of King’s College London. After birth, pups remained with the dam until weaning on day 21. Animals were kept on a 12 h light/dark cycle (lights on from 07:00 to 19:00) under a constant temperature (22 ± 2°C) and humidity (55% ± 5) with access to food and water *ad libitum*.

All animal experiments were performed under appropriate Home Offices licenses. Experimental protocols were in accordance with the international guidelines for the care and use of laboratory animals (EU Directive 2010/63/EU for animal experiments) and the Animal Research: Reporting of *in vivo* Experiments (ARRIVE) criteria. All animal monitoring was performed during daytime. Criteria for discontinuation of the experiments and humane end points were evaluated throughout the experiments. The taxonomy suggested by the International Association for the Study of Pain was used (Merskey and Bogduk, 1994).

### Materials

AAV9-GCamP6s at a titer of ≥1 × 10^1^^3^ viral genomes/ml was obtained from Addgene (Watertown, MA, USA). CFA (F5881, Sigma, Manchester, UK) was stored at 4°C. rBoNT/A1 (IPN10260, Ipsen Bioinnovation, UK) was freshly dissolved prior to injection at 5 pg per 20 μl in vehicle solution based on a gelatine phosphate buffer (GPB) composed of gelatine 0.2% and disodium hydrogen phosphate 50 mM, at pH 6.5 (orthophosphoric acid).

### Preparation of rBoNT/A1

rBoNT/A1 gene synthesis, expression of *E. coli*, and purification methods have been described previously (Hooker et al., 2016). Briefly, the BoNT/A1 protein sequence was back-translated and codon-optimized for expression in *E. coli*. The DNA sequence was synthetized in two parts and subsequently combined to create a coding sequence for a full-length neurotoxin. Repeated batches of consistent material were produced and showed equivalent activity to native BoNT/A1 in several biochemical assays (Hooker et al., 2016). rBoNT/A1 was shown to be comparable to native BoNT/A1 *In vitro* in inhibiting the acetylcholine release by cleaving SNAP25 in the rat embryonic spinal cord neurons and, *ex vivo*, in the mouse phrenic nerve hemidiaphragm assay. *In vivo*, equipotent effects of rBoNT/A1 and native BoNT/A1, injected intramuscularly, were demonstrated, and had similar onset and duration of action in the rat digit abduction assay in mice and rats (Périer et al., 2021). Furthermore, rBoNT/A1 injected into the gastrocnemius muscle of the rat, dose-dependently reduced muscle weight and volume, induced myofiber atrophy and cleaved SNAP25 at the neuromuscular junction and the peripheral nerves.

### Measurement of Mechanical Allodynia With Von Frey Filaments: Experimental Design and Behavioral Assessment

**Figure 1:**
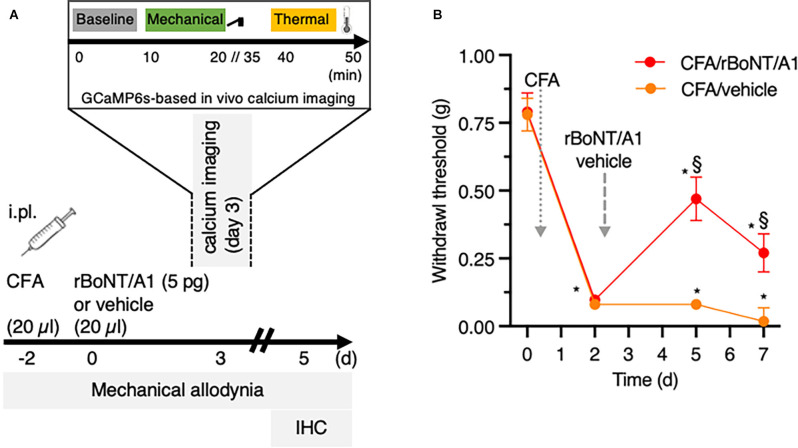
rBoNT/A1 ameliorates mechanical allodynia in CFA-evoked local hind paw inflammation in mice. **(A)** Schematic representation of the experimental protocol used for *in vivo* calcium imaging, behavioral and histology experiments. CFA and rBoNT/A1 or vehicle (gelatine phosphate buffer; GPB) were injected on day -2 and day 0, respectively, followed by behavior measurements on day 3 and 5. *In vivo* calcium imaging was performed on day 3. Recordings at baseline were followed by mechanical and thermal stimulation. On day 5, tissues were collected for histological analysis. **(B)** Mechanical allodynia (g) was measured by von Frey filaments. Two-way ANOVA, *post hoc* Tukey. *(within groups to baseline) and §(across groups): *p* < 0.05. *N* = 10/group.

Mice were habituated to the facility and the experimental setup, before being assessed for mechanical sensitivity by measurement of withdrawal threshold using calibrated von Frey filaments applied to the plantar surface of the left hind paw at baseline, on three consecutive days (Day-4, Day-3, and Day-2; Tough-Test Sensory Evaluator; Scientific Marketing Associates; Barnet, UK). The mean of Day-3 and Day-2 were considered as baseline (Figure 1A). Mechanical sensitivity was also tested on day 0 (before rBoNT/A1 or vehicle treatment) and on days 3 and 5. A series of graduated von Frey filaments (0.07, 0.16, 0.4, 0.6, and 1 g) were applied in sequence with a protocol of 1 s on/1 s off, repeated 10 times. Each filament was applied perpendicularly to the center of the ventral surface of the paw until it bent slightly. The force applied to the hind paw of the animal to induce 5 responses out of 10 trials was recorded as the paw withdrawal threshold.

Mice were injected intraplantarly with 20 μl of CFA (1.5 mg/ml) two days before rBoNT/A1 or vehicle treatment (day-2; Figure 1A). On day 0, animals received intraplantar injections of either rBoNT/A1 (5 pg/mouse) or vehicle (GPB) into the same ipsilateral hind paw (*n* = 10/group). All intraplantar injections were made at 20 μl volume and performed with a 30 G needle under a brief isoflurane anesthesia. Both experimental groups, rBoNT/A1- and vehicle-injected animals, were kept in the same cage. The experimenter was blinded to treatment. At the end of the experiment, mice were euthanized with CO_2_.

### *In vivo* Calcium Imaging in DRG Neurons

#### GCaMP6s Treatment

At the age of 2–5 days, pups received intraplantar injections of 5 μl AAV9-GCaMP6 into the left hind paw (ipsilateral site of rBoNT/A1 injection and *in vivo* imaging; see below). After reaching about 18–30 g bodyweight, animals were randomly allocated to their experimental groups. Animals were closely monitored throughout the experiment. If the bodyweight decreased below 20% of the initial weight, mice were euthanized. Depending on the litter size, the authors aimed to use siblings of the same sex within the same experimental day. At the end of the imaging session mice were humanely euthanized with an overdose of pentobarbital.

### Animal Preparation for Imaging

In line with the behavioral setup, adult mice, treated with AAV9-GCaMP6 as neonates, received intraplantar injections of 20 μl CFA (1.5 mg/ml) into the left hind paw on day-2 (Figure 1A). Two days later (day 0), either rBoNT/A1 (*n* = 5) or vehicle (GPB; *n* = 7) were applied to the same site. An additional group of mice with the neonatal history of AAV9-GCaMP6 treatment (*n* = 6) received intraplantar injections of phosphate buffered saline (PBS; 20 μl) on day-2 and injections of vehicle (GPB) on day 0. On day 3 calcium transients were recorded in the L4 DRG neurons containing most of the somata of the peripheral terminals of the plantar surface of the murine hind paw. After induction of anesthesia with high Vol% Isoflurane in an induction box (Uno BV) the mice were, throughout the experiment, spontaneously breathing 1–1.5 Vol% isoflurane mixed with oxygen in a semi-closed anesthesia aperture at a flow rate of 1 l/min *via* a nose cone. Body temperature was controlled by a heat mat and rectal thermometer (FHC) and adjusted to initial levels, around 36°C. Eyes were kept moist with artificial tears and the mice received a subcutaneous injection of isotonic saline (10 μl/g bodyweight). After shaving the back, the skin above the vertebral column was incised from the lower thoracic to the sacral level. The rostral part of the vertebral column was clamped in a custom-made frame with forceps to decrease movement artifacts evoked by breathing excursions and positioned in a more lateral recumbent position to placing the DRG in a more horizontal position (Chisholm et al., 2018). On the left-hand site, around vertebrae L4, the fasciae and muscles covering the lateral processus were removed. With small rongeurs, the lateral part of the bone was gently cracked apart to uncover the L4 DRG. To prevent drying, tissue was coated with a thin layer of low melting agarose. The left hind paw was retroflexed, so the plantar surface was pointing upwards. We observed that animals were more likely to die under isoflurane anesthesia after receiving rBoNT/A1 due to weakness and quality of imaging decreased over time, e.g., by bleaching and drying of the tissue.

### Stimulation Protocol

To evaluate the sensitivity of peripheral sensory nerve endings innervating the corresponding, ipsilateral hind paw, neuronal activity in the DRG neurons was recorded. After sampling baseline activity of the left L4 DRG neurons for 10 min, the left hind paw was stimulated first mechanically and then, after an extended interval, thermally (Figure 1A).

**Figure 2:**
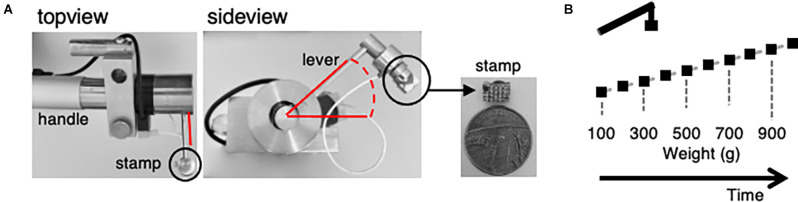
A custom-made electronically controlled mechanical stimulator and the mechanical stimulation protocol for *in vivo* calcium imaging. **(A)** Photos of the electronically controlled mechanical stimulator and its stamp. Top view showing the lever attached to the handle, sideview giving insight into the rotation angle of the lever highlighted in red and the stamp attachment (black circle). Enlarged picture of the stamp in comparison to a one penny coin. **(B)** Illustration of the mechanical stimulation protocol. Weight was increased from 100 g (0.09 N) to 1,000 g (0.9 N) in 100 g increments every 60 s. Stimulus duration was about 1 s.

To assess the mechanical sensitivity, a custom-made, electrically-controlled stamp device was established (Figure 2A). The mechanical stimulator consisted of an electric motor that controls a lever *via* pinions, stored in a handle. The top-view shows a part of the handle and the lever attached in a 90° angle. The sideview depicts the ball joint and gives the range of motion of the lever, about 23°. A little stamp with 6 × 4 blunt pins, about 2 mm in height and 0.1 mm spaced apart, measuring about 6 × 4 mm, magnitudes smaller than a UK 1 pence coin, was magnetically attached to the distal end of the lever. Current applied to the electrically controlled lever was converted into movement. The stamp was positioned 1.5 mm above the plantar surface of the left hind paw without contact to the skin. Continuous contact of the stamp with the glabrous skin was avoided to prevent constant stimulation of the innervating nerve endings. Acceleration was neglectable. Calibration of the overall force of the plane surface of the lever on a kitchen scale revealed a minimum of 100 g (approx. 0.09 mN per pin). Application of the minimum pressure was followed by a 100 g increments, until reaching 1,000 g maximum (approx. 0.9 mN per pin) after one-minute intervals before applying the next stimulus (Figure 2B). Thermal stimuli were applied by a peltier device (TSAII, Medoc; Ramat Yishay, Israel) placed on the plantar surface of the concomitant, left hind paw. The temperature of the 16 × 16 mm probe was increased by ramps of 4°C/s from baseline (32°C) to 40°C, 45°C, and 50°C, respectively. Each heat stimulus was applied for 8 s followed by 60 s baseline before the next stimulus.

### Data Acquisition

Data were acquired with a 10-fold dry objective attached to an upright Eclipse Ni-E FN confocal/multiphoton microscope (Nikon UK, Branch of Nikon Europe B.V., Amsterdam, The Netherlands) using a 488-nm Argon ion laser line. GCaMP signal was collected at 500–550 nm. Time series recordings were taken with an in-plane resolution of 512 × 256 pixels at an acquisition speed of 3–4 Hz. Data were saved with date and experiment number to encrypt the experiments and to impede a biased analysis.

### Tissue Analyses

In line with behavior and imaging experiments, the same treatment protocol was used for tissue analysis in an independent cohort of 13 mice. On day five, CFA-exposed mice that were injected with either rBoNT/A or vehicle (*n* = 5/group) were euthanized, and tissues were collected for analyses. A group of untreated, untreated animals (*n* = 3) was added as absolute controls. The injected footpad (three tissues/section/animal), lumbar vertebrae including DRGs and the lumbar spinal cord (at least four tissues/section/animal) were fixed in 10% formalin for 48 h, decalcified and embedded in paraffin blocks. Histological slides were prepared. Footpad tissue sections were stained with haematoxylin and eosin (H&E) for standard histopathologic examination and evaluation of the inflammatory process. Severity of lesions were classified using the following standard grading system: 0 (no lesions), 1 (minimal), 2 (mild), 3 (moderate), 4 (severe), 5 (marked).

The immunohistochemical staining for SNAP25 and cleaved SNAP25 were performed using a standard avidin-biotin-peroxidase procedure as previously described (Périer et al., 2021). After a heat-induced epitope retrieval step, endogenous peroxidases were blocked for 10 min in a 3% H_2_O_2_ solution in a TBS buffer. The sections were incubated with a non-commercial primary rabbit polyclonal antibody (EF14007, Ipsen Innovation, Les Ulis, France) which is specific for the cleaved form of SNAP25 by BoNT/A only or with an antibody directed against the N-terminal part of SNAP25, thus recognizing both SNAP25 full and cleaved forms (SYSY 111 008, Synaptic Systems, Göttingen, Germany; overnight incubation). Sections were then incubated with a biotinylated secondary antibody for 30 min (anti-rabbit IgG, Vector Laboratories, Burlingame, CA, USA), followed by a 30-min incubation with an amplification system (avidin-biotin) coupled to horseradish peroxidase (Vector Laboratories, Burlingame, CA, USA). Finally, sections were incubated for 5 min with a 0.02% diaminobenzidine solution (DAKO, Carpinteria, CA, USA). To counterstain, haematoxylin was used, and the slides were imaged with a light microscope.

For the quantification of the amount of cleaved SNAP25 in muscles, the number of stained neuromuscular junctions (NMJ), intensity of staining, the presence of staining in terminal nerve ending and larger intramuscular nerves was used as previously described (Périer et al., 2021). In the spinal cord, the intensity and density of cleaved SNAP25 positive nerve endings was graded as follows: 1 (minimal), 2 (mild), 3 (moderate), 4 (marked) on the 4 most intensely stained spinal cord sections; a cumulative score (0–16) was then calculated for each animal in the ipsilateral dorsal and ventral horns.

### Statistical Analysis

For behavioral tests, the minimum number of animals to reach statistical significance was estimated by an *a priori* power analysis (Faul et al., 2009). For the statistical analysis, InVivoStat^1^ (Clark et al., 2012) and Prism 7 for Mac OS X (GraphPad Software, San Diego, CA) were used to calculate a two-way repeated measures ANOVA followed by Tukey *post hoc* multiple comparison after normality check. Values given in the results section are the *P*-values for interaction within or across groups as indicated (*P* < 0.05).

Prior to statistical analysis of the *in vivo* calcium imaging experiments a mixture of published codes (Pachitariu et al., 2016) and custom written scripts in Python (python.org) and Matlab 2019a (MathWorks) were used to process the data. After exporting the nd2 files obtained during recording with Nikon Elements software (Nikon) as tiffs, data were loaded into Suite2p, a Python script to identify regions of interest (ROIs; Pachitariu et al., 2016). Threshold parameters were kept constant throughout analysis of all experiments. The results were then imported into Matlab to perform further analysis. In accordance with our previously performed analysis of *in vitro* calcium imaging (Martin et al., 2018), data were normalized to baseline fluorescence after correction for the neuropil, reflecting data points out of focus and scattered fluorescence from nearby processes (e.g., axons, dendrites, synaptic boutons) as well as subtraction of averaged baseline values. Then, cells with high initial calcium concentrations as well as those cells not returning to baseline 3 min after maximum stimulation were excluded. To compare the magnitude of responses for each stimulus, the area under curve (AUC) was calculated in each individual neuron for a 15 s period after stimulation, including the peak hight. The interval started 20 frames before and ended 35 frames after the stimulus. Only cells reaching at least calcium concentrations 25% above their baseline AUC at the maximum stimulus were considered as positive responders. Later, the size of the AUC was correlated with the different mechanical stimuli used. When the correlation coefficient was >0.7, suggesting a 70% probability of linear correlation, cells were counted as nociceptors and were therefore included into subsequent statistical analysis. Event counts were conducted on deconvoluted data, an analysis implemented in Suite2p (Pachitariu et al., 2016; Friedrich et al., 2017). The deconvolution of calcium transients into spikes was a tool implemented into the Suite2p analysis program. After extracting the information, the data were processed to generate a 1 or 0 code.

For histopathology and cleaved SNAP25 staining analyses, the mean of CFA/vehicle group was compared to the one of CFA/BoNT group using non-parametric Mann-Whitney test.

Statistical tests, indicated in the figure legends, were performed with GraphPad Prism (San Diego, CA, USA). Equally sized experimental groups were planned prior to the study aiming for eight mice per group. However, in the BoNT group mice were more vulnerable to anesthesia and therefore died early. To keeping the group size similar, fewer experiments than planned were conducted in the CFA plus vehicle and the control group. Throughout the analysis, data obtained in the same mice were used. Here, due to technical and methodological issues, the animal numbers included in each analysis varies. Even though we are aware of this problem, increasing group sizes to even out the statistical analysis would fail to prove that hypothesis that BoNT reduces CFA-mediated sensitization of peripheral nerve endings.

## Results

After obtaining baseline parameters on mechanical sensitivity by von Frey measurements, CFA was injected into the footpad (Figure 1B). The withdrawal thresholds dropped from 0.8 g at baseline by factor of 10 2 days later. Three days after, rBoNT/A1 or vehicle (GPB) application into the inflamed paws, the withdrawal thresholds in the rBoNT/A1-treated group increased significantly by a factor of five compared to mice receiving vehicle treatment only. Those thresholds remained at low levels. Changes in withdrawal thresholds in the CFA/ rBoNT/A1 group declined on day five by 57%, still being 15-times above the control group.

To investigate the inhibition of inflammatory, mechanically evoked hypersensitivity in mice by rBoNT/A1 a custom made electrically controlled mechanical device was introduced (Figure 2A). Photos show the device (Figure 2). The top-view displays the handle and the rectangularly attached lever. The sideview depicts the ball joint and gives the range of motion of the lever. *via* a little stamp with blunt pins forces were applied to the footpad. Starting with a stimulus of 100 g (about 0.09 mN per pin) for a minimum contact time (approx. 1 s), the weight pushing onto the specimen was increased every minute by 100 g until reaching 1,000 g (0.9 mN per pin; Figure 2B). During the interval, the stamp was distanced from the skin.

**Figure 3:**
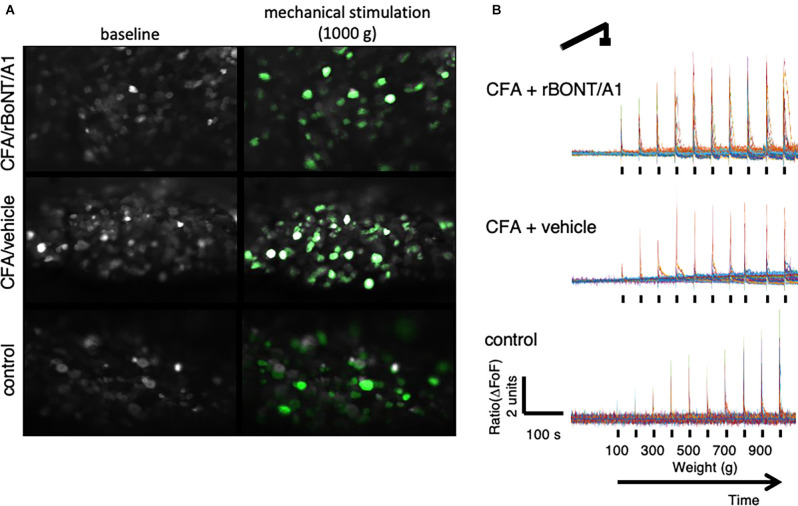
Peripherally applied pressure to hind paws evokes calcium transients in dorsal root ganglion (DRG) neurons *in vivo*. **(A)** False color images of GCaMP6s-labeled DRG neurons *in situ* at baseline and after maximum mechanical stimulation (1,000 g/0.9 N). **(B)** Traces depict calcium transients normalize to baseline (ΔFoF) in individual neurons from mice treated with CFA and rBoNT/A1 (top), CFA and vehicle (GPB; middle), or control (PBS; bottom) upon mechanical stimulation as indicated over time.

Stills of DRG neurons extracted from the recordings in mice receiving CFA/rBoNT/A1 (top), CFA/vehicle (middle), or PBS (bottom), illustrate the increase in fluorescence signal upon maximum mechanical stimulation compared to baseline fluorescence in individual DRG neurons (Figure 3A). The fluorescence traces (ΔFoF) depict corresponding examples of intracellular calcium fluctuations upon mechanical stimulation over time (Figure 3B).

**Figure 4:**
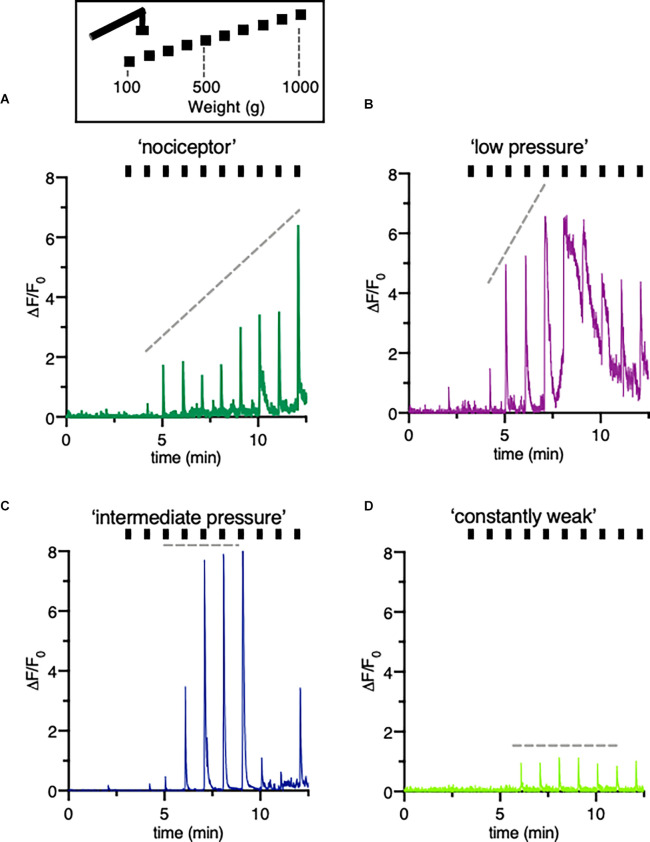
Classification of different type of DRG neurons responsive to increasing pressure. **(A–D)** Schematic drawing of the mechanical stimulation protocol and example trace indicating a variety of fluorescence responses taken from individual neurons of a mouse treated with vehicle only. The hindpaw was stimulated with increasing pressure started at 100 g to a maximum of 1,000 g. Different patterns of changes in intracellular calcium concentrations reflect distinctive cell types, here called “nociceptors” **(A)** “low pressure” responders **(B)** “intermediate pressure” responders **(C)** and “constant weak” **(D)** responders. Ticks correspond to mechanical stimulation protocol.

Forces from 100 g (0.09 mN) to 1,000 g (0.9 mN) were applied over 10 min. It appeared that the response patterns which lined up with the corresponding mechanical stimulus were different in mice treated with PBS in comparison to mice with an inflamed hind paw. To identify distinctive cell types, signals were sorted by different response patterns (Figure 4). The analysis of the individual traces confirmed a group recognized as “nociceptors” (Figure 4A).

**Figure 5:**
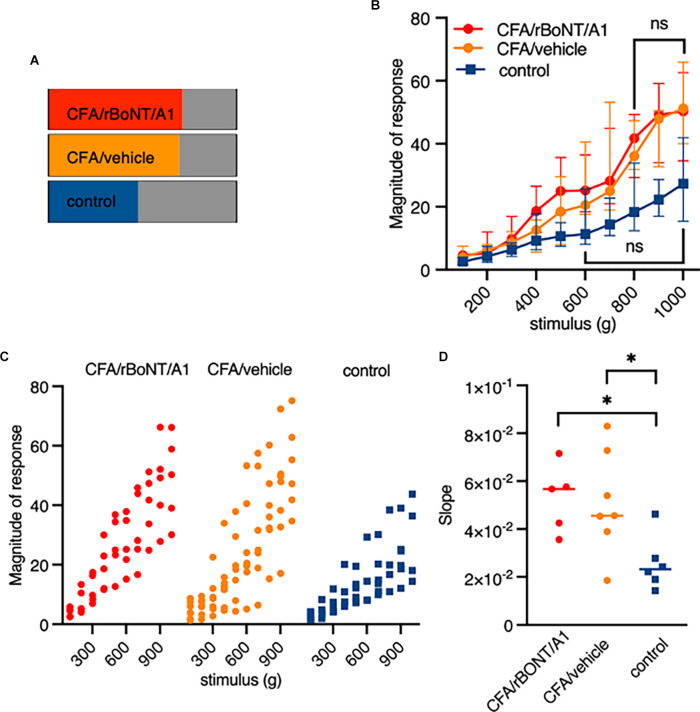
Pressure responsive “noiceptors” are sensitized by inflammation but not inhibited by rBoNT/A1 on a cellular level. **(A)** Percentage of identified “nociceptors” under indicated conditions. **(B)** Magnitude of responses over stimulus in the three different treatment groups as median plus interquartile range. Significant difference between 100 and 700 g in CFA/rBoNT/A1 and CFA/vehicle as well as 100–500 g in control (PBS) group compared to maximum stimulus. Indication in graph highlights first stimulus that is not significantly different to 1,000 g. No difference in between the CFA/rBoNT/A1 and CFA/GPB treatment groups. Mixed-effect analysis, *post hoc* Tukey. *p* < 0.05. **(C)** Magnitude of responses in individual animals presented as data clouds over stimulus grouped by treatment. **(D)** Comparison ofresponse/stimulus slopes from individual mice calculated from the median in Figure 4B. Welch ANOVA, *post hoc* Dunnett. ^*^p < 0.05. *n*(CFA/rBoNT/A1) = 5; *n*(CFA/vehicle) = 7; *n*(PBS) = 6. ns, not significant.

**Figure 6:**
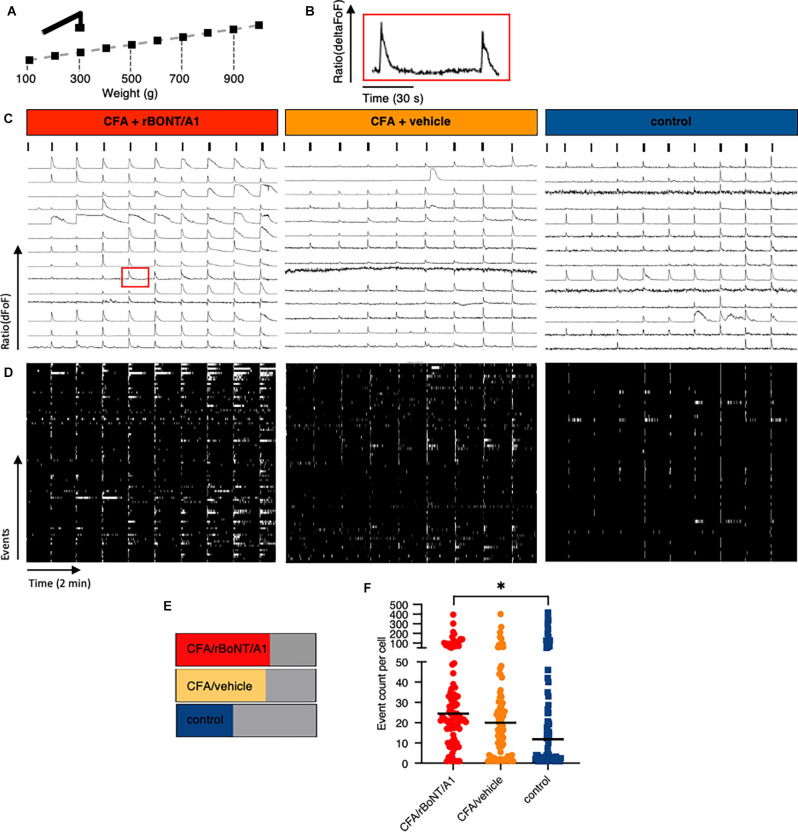
Prolonged discharge of DRG neurons after mechanical stimulation in mice with local CFA-provoked inflammation uncovered by *in vivo* calcium imaging. **(A)** Schematic drawing of the mechanical stimulation protocol corresponding to the traces shown below. **(B)** Enlarged trace of a calcium transient taken from **(C)** (CFA/rBoNT/A1, 500–600 g, 9th cell, red square). **(C)** Example traces of fluorescence changes upon mechanical stimulation over time in any DRG neuron of three different mice treated as indicated. Ticks indicate mechanical event as shown in **(A)**. **(D)** Raster plots of deconvoluted data of calcium events corresponding to individual calcium traces shown in **(C)**. **(E)** Percentage of cells responding to mechanical stimuli under different treatment conditions as indicated. **(F)** Plot of summed deconvoluted events per cell and median taken from animals with indicated treatments. *n*(CFA/rBoNT/A1) = 189; *n*(CFA/vehicle) = 175;*n*(control) = 187 neurons of *n* = 3 mice per group. Kruskal-Wallis ANOVA, *post hoc* Dunn. ^*^p < 0.05.

Constantly rising force applied to the peripheral nerve endings in the footpad of the mice evoked a linear correlating increase in fluorescence intensities. In other neurons of the same DRG, either low and intermediate pressure already evoked a maximum response (Figures 4B,C) or the signal was constantly weak regardless the forces applied (Figure 4D). Counting the number of mechanically activated “nociceptive” neurons uncovered that 67% and 64% of all neurons respond to mechanical stimuli after induction of local, peripheral inflammation with CFA treated with rBoNT/A1 or vehicle, respectively (Figure 5A). In the group treated with PBS, approximately 40% of the “nociceptive” neurons responded to pressure. The averaged responses of the “nociceptors” were increased upon mechanical stimulation in mice with local CFA-evoked inflammation (Figure 5B). Differences were significant at high pressure, starting at 700 g (*p*_(CFA/rBoNT/A1)_ = 0.44 and *p*_(CFA/vehicle)_ = 0.04). Throughout the experiments, the averaged magnitude of responses was about two-fold increased under inflammatory conditions. Co-application of rBoNT/A1 failed to alter CFA-evoked responses. A detailed analysis of the data clouds unmasks an increased variance under inflammatory conditions (Figure 5C). The slopes of the rising responses in individual animals were significantly different between the CFA group and the control group (*p* = 0.04; Figure 5D). After identifying different subsets of neurons, fluorescence signals were analyzed in more detail. Randomly picked, individual fluorescence traces from DRG neurons of mice treated with CFA ± rBoNT/A1 or vehicle alone are displayed upon mechanical stimulation (Figures 6A–C).

Mechanical withdrawal thresholds in inflamed paws of freely moving mice were significantly ameliorated by rBoNT/A1. To evaluate whether the discharge in any mechanosensitive neuron was affected, we analyzed the different response patterns of all individual neurons activated by mechanical stimulation. High magnification analysis of the plotted traces uncovered that prolonged, unsteady calcium fluctuations were detected in some but not all neurons before the subsequent stimulus (Figure 6B). This observation prompted to the analysis of deconvoluted calcium events (Figure 6D). Raster plots uncovered that calcium fluctuations were prolonged in some individual neurons after mechanical stimulation. The number of cells with prolonged activity in CFA treated animals remained unaffected regardless the treatment with rBoNT/A1 or GPB but increased by 25% in comparison to the control (Figure 6E). A quantitative comparison of the activity of individual neurons in different animals verified that the median of the summed events per cell increased from 11.8 to 20 and 24.5 under inflammatory conditions (Figure 6F). However, also in this in-depth analysis, rBoNT/A1 failed to alter the median of in CFA-treated animals compared to the neurons of mice receiving CFA and vehicle treatment only (*p* = 0.07). Hence, rBoNT/A1 had no effect on mechanically evoked responses under inflammatory conditions.

**Figure 7:**
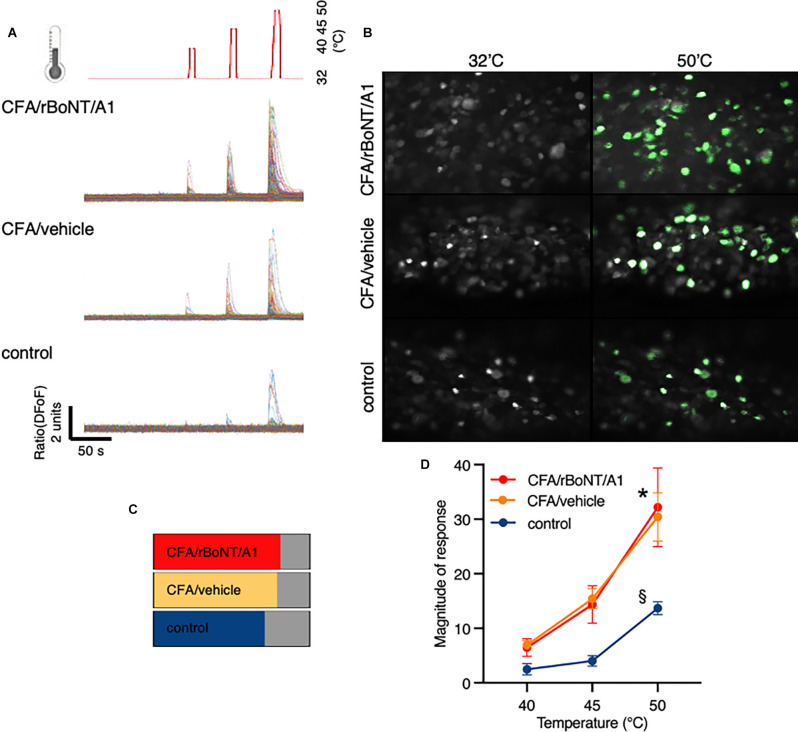
rBoNT/A1 fails to inhibit calcium transients in the DRG upon peripheral heat stimulation. **(A)** Schematic drawing of the thermal stimulation protocol in line with traces below depicting calcium transients normalize to baseline (ΔFoF) in individual neurons from mice treated with CFA and rBoNT/A1 (top), CFA and GPB (vehicle; middle), or vehicle alone (bottom) over time. Baseline temperature was set to 32°C. To apply stimuli, heat was increased to 40°C, 45°C, and 50°C for 8 s each. In-between the stimuli, temperature returned to baseline. **(B)** False color images of GCaMP6s-labeled DRG neurons *in situ* at baseline and after noxious heat stimulation (50°C) from mice treated with CFA and rBoNT/A1 or CFA and vehicle (GPB) or vehicle alone. **(C)** Percentage of neurons responding to noxious heat under conditions as indicated. **(D)** Magnitude of responses upon heat stimulation with 40°C, 45°C, and 50°C in treatment groups as indicated. *N* = 4 for CFA/rBoNT/A1, *n* = 5 for CFA/vehicle and *n* = 5 for control (PBS). Mean ± SEM. Mixed-effect analysis, *post hoc* Tukey. *p* < 0.05. *(across groups): CFA plus rBoNT/A1 or vehicle vs. control; §(within groups) compared to 40°C.

To assess changes in GCaMP6s fluorescence in DRG neurons upon thermal stimulation, the linked hind paw was stimulated every minute for eight seconds by increasing heat stimuli using a ramp protocol. Temperatures peaks were set to 40°C, 45°C, and 50°C, respectively after recoding baseline fluorescence at 32°C (Figure 7A). Plotted traces depict the rising fluorescence changes over time (ΔFoF) in line with the applied temperatures. Pictures from the top to the bottom show corresponding false-colored DRG neurons from mice who received CFA and rBoNT/A1, CFA and vehicle or PBS (Figure 7B). Fluorescence brightness changed dramatically upon noxious heat stimulation (50°C) compared to baseline. The pictures suggest an increase in numbers of responding neurons upon thermal stimulation depending on the local treatment. About 80% of the neurons responded to thermal stimuli under inflammatory conditions regardless the co-treatment with rBoNT/A1 or vehicle. Statistical analysis unmasks that the proportion of responding cells after CFA was only increased by less than 10% compared to control (Figure 7C). Under given treatment conditions, CFA sensitization evoked a significant increase in responses at noxious temperatures compared to the group without inflammatory responses (Figure 7D; PBS vs. CFA/BoNT: *p* = 0.007 or CFA/vehicle: *p* = 0.01). Responses in DRG neurons of mice treated with CFA significantly increased at noxious 50°C in comparison to 40°C (both: *p* < 0.001). rBoNT/A1 failed to inhibit these responses (*p* > 0.99). In the PBS group, changes in the magnitude of response were not significantly different at any time. Non-noxious temperatures (40°C) barely changed the calcium influx in DRG neurons regardless the treatment. Hence, the data confirmed that CFA sensitizes the terminal nerve endings resulting in an upregulated response upon hot temperature stimulation but rBoNT/A1 has no impact.

In the footpad of untreated mice uncleaved SNAP25 was identified in nerve fibers terminating in the epidermis (Supplementary Figure 1A), surrounding apocrine glands (Supplementary Figure 1B), in peripheral nerve bundles (Supplementary Figure 1C), in nerves innervating arteries (Supplementary Figure 1D) and at neuromuscular junctions (Supplementary Figure 1E). Cleaved SNAP25 (c-SNAP25), on the other hand, was not detected in any of the samples taken from mice treated with CFA/rBoNT/A1 (Supplementary Figures 1F–I) except at the neuromuscular junctions of the small muscles located in the footpad (Supplementary Figure 1J). Nothing was labeled by the isotype antibody, confirming the specificity of the staining (Supplementary Figures 1K–O). Quantification of the labeling showed significantly higher levels of c-SNAP25 in the neuromuscular junctions of CFA/rBoNT/A1-treated animals compared to CFA/vehicle-treated mice and absolute controls (Supplementary Figure 1P).

**Figure 8:**
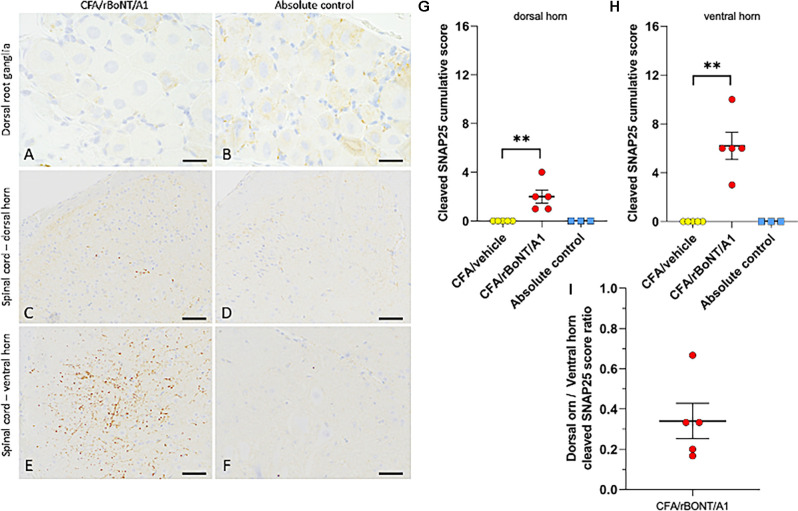
Cleaved SNAP25 in the dorsal root ganglion (DRG) and the lumbar spinal cord. **(A,B)** Representative images of the dorsal root ganglion (DRG) and **(C–F)** of the spinal cord of mice treated with CFA/rBoNT/A1 or in untreated controls. Cleaved SNAP25 was absent in the DRG (despite higher background staining), while being present in the nerve ending-like structures in the ipsilateral dorsal **(C)** and ventral **(E)** horns of the lumbar spinal cord of CFA/rBoNT/A1-treated animals, but not in absolute controls. Bars **(A,B)** (20 μm), **(C–F)** (50 μm). **(G–I)** Quantification of cleaved SNAP25 in the lumbar spinal cord in CFA/rBoNT/A1-treated animals compared to CFA/vehicle and absolute controls. c-SNAP25 staining was moderate in the dorsal horn **(G)** and high in the ventral horn of the spinal cord **(H)** while being absent in CFA/vehicle-treated animals and in absolute controls. **(I)** The dorsal horn/ventral horn ratio of c-SNAP25 (< 1) indicates higher targeting of ventral vs. dorsal horn by rBoNT/A1. *N* = 5/group (CFA/rBoNT/A1, CFA/vehicle) and *N* = 3 (absolute control). Mean ± SEM. ^**^p < 0.01 CFA/rBoNT/A1 vs. CFA/vehicle; Mann-Whitney test.

Specifically focussing on neuronal tissue, c-SNAP25 was lacking in DRG neurons but was identified in the corresponding ipsilateral dorsal and ventral horns in animals treated with rBoNT/A1 (Figures 8A,C,E). There was no evidence of c-SNAP25 in untreated animals (Figures 8B,D,F). Quantification of the stainings revealed a significant increase of c-SNAP25 in the spinal cord that was modest in the ipsilateral dorsal horn (Figure 8G) and high in the ipsilateral ventral horn (Figure 8H) of the rBoNT/A1-injected animals in comparison to CFA/vehicle and untreated mice. This was also confirmed by the dorsal horn/ventral horn c-SNAP25 score ratio being inferior to 1 (Figure 8I).

H&E staining of the foot pad of mice clearly demonstrated that inflammation was present in CFA-treated animals (Supplementary Figures 2A–C), while being absent in controls (Supplementary Figures 2D,E). Immune cells infiltrated the site of lesion and formed foamy vacuoles containing a foreign substance, most likely CFA (Supplementary Figures 2B,C) but were absent in controls (Supplementary Figure 2E). The overall inflammation score in the CFA/rBoNT/A1 group equaled the CFA/vehicle group (Supplementary Figure 2F) even though, the number of neutrophils was slightly lower in inflamed tissue of rBoNT/A1- than in vehicle-treated mice. This trend failed to reach statistical significance (*p* = 0.48; Supplementary Figure 2G).

## Discussion

Here, we have used an *in vivo* optical imaging method to investigate whether analgesic activity of rBoNT/A1 was mediated by changes in the activity of peripheral nociceptors in a model of inflammatory pain. To achieve this, we simultaneously monitored activity in cell bodies of hundreds of sensory neurones located in the ipsilateral DRG of rBoNT/A- vs. vehicle-treated animals with CFA-evoked local inflammation. Peripherally evoked signals are transmitted *via* the afferent axon to the soma of the pseudo-unipolar DRG neurons. *In vivo* imaging of DRG neurons is used as a proxy for events at the terminal nerve endings. Previous reports show an enhanced action potential firing upon local thermal stimulation of nerve terminals in inflamed paws of rodents (Djouhri et al., 2006). We found that CFA-mediated inflammation is also associated with increased calcium influx in the DRG neurons. In comparison to electrophysiological recordings, *in vivo* calcium imaging can be used for screening by recording signals of many neurons at the same time. To investigate whether the site of action of BoNT exerts from the periphery, we evaluated if upstream calcium signal responses in L4 dorsal root ganglion neurons (DRGs) were inhibited after local, peripheral injection of rBoNT/A1 into inflamed paws. Recorded calcium fluctuations linked to peripheral stimulation of the nerve terminals prompt to a peripheral mode of action while a lack of changed responses rather implicates a more upstream site of action of BoNT, which is likely to be in the spinal cord.

The primary purpose of this study was to ask if botulinum toxin prevents the transmission of the peripherally evoked signal in sensory nerve endings to their soma in the DRG in an inflammatory model of pain in mice. BoNT has been used in several pre-clinical and clinical trials to reduce inflammatory and neuropathic pain (Yoo et al., 2014; Finnerup et al., 2015; Egeo et al., 2020). Behavioral studies in both, humans and rodents, suggest that BoNT suppresses hypersensitivity when applied locally into inflamed subcutaneous tissue (Shi et al., 2020). We confirmed that withdrawal reflexes upon mechanical stimulation were diminished in rBoNT/A1-treated animals in comparison to vehicle-treated controls. In line with our data, an investigation of the inhibitory profile of BiTox, a modified botulinum toxin, on CFA-evoked inflammatory pain revealed a reduction in mechanical hypersensitivity in rats while thermal responses remained unchanged (Mangione et al., 2016; Maiarù et al., 2018). In our study, the antinociceptive effect of rBoNT/A1 is not mediated by a reduced inflammation as proven by similar levels of inflammatory responses and neutrophils in rBoNT/A1- and vehicle-treated animals with local CFA-provoked hind paw inflammation.

The principal mode of action of BoNT/A1 is the cleavage of SNAP25, a component of the SNARE complex necessary for the calcium-mediated vesicular release of neurotransmitters and other proteins. Several studies have confirmed that BoNT/A1 reduces the CGRP release in migraine, its acknowledged pathophysiological mechanism, as well as the release of substance P, one of the major pain-related neuropeptides (Montecucco et al., 1996; Purkiss et al., 2000; Durham et al., 2004; Matak et al., 2017; Joussain et al., 2019). We endorse previous findings that the terminal nerve endings remain intact after local rBoNT/A1 injection (Paterson et al., 2014). The chilli receptor transient potential receptor channel vanilloid-type 1 (TRPV1) is one of the principal peripheral pain sensors responding to noxious heat. After BoNT/A1 treatment, the plasma membranes abundancy of TRPV1 is diminished on dorsal root ganglia (Xiao et al., 2013; Li and Coffield, 2016; Meng et al., 2016; Cao et al., 2017; Fan et al., 2017). A study on cultured DRG neurons showed a reduction in slowly adapting currents upon mechanical stimulation in cells treated with BoNT/A (Paterson et al., 2014). Conversely, in our study calcium transients in DRG neurons distant from the stimulation site were not reduced. Also, our data did not confirm a decreased responsiveness to thermal stimuli after rBoNT/A1 application although our dosing regime was effective.

In addition to the peripheral effects, it has been shown that BoNT suppresses the neurotransmitter release in primary synapses in the spinal cord (Kim et al., 2015; Matak et al., 2017). There is evidence that unilateral peripheral injection of rBoNT/A1 into a hind paw of rats decreases hypersensitivity thresholds on the contralateral site in a model of chemotherapeutic-induced polyneuropathy (Favre-Guilmard et al., 2009, 2017). Our data imply that the analgesic effect rather might take place in the central nervous system consistent with the hypothesis that the toxin is retrogradely transported to its place of action in the spinal cord (Marinelli et al., 2012; Papagiannopoulou et al., 2016). This is strengthened by our data revealing the total absence of c-SNAP25 in the DRGs of rBoNT/A1-treated animals with CFA inflammation and its simultaneous presence in the lumbar spinal cord. After intraplantar administration of rBoNT/A1, high quantities of c-SNAP25 were observed in the ventral horn and low to moderate levels in the dorsal horn. These data confirm the results in the rats where the effects were abolished by colchicine, a neuronal transport blocker (Matak et al., 2012). Higher targeting of the ventral horn is consistent with the intraplantar route of administration that triggers strong rBoNT/A1 effects in skeletal muscles, as seen in c-SNAP25 staining of the neuromuscular junctions in injected footpads. However, presence of low levels of SNAP25 cleavage in the dorsal horn suggests the targeting of sensory nerves that might be sufficient to reduce nociceptive behavior.

We studied intracellular calcium changes upon mechanical stimulation using a novel custom-made stimulator. The new device allowed to repetitively apply defined levels of pressure onto the inflamed hind paws of our mice. We discovered that, in a subset of cells, increased pressure linearly correlated with the fluorescence intensity changes. These data confirm responses obtained in behavioral studies in rodents testing mechanical hypersensitivity under inflammatory conditions. However, mechano-sensitization evaluated by electrophysiological recordings is controversial. While some groups identified sensitized fibers, others missed these findings (Andrew and Greenspan, 1999; Koltzenburg et al., 1999; Bishop et al., 2010; Lennertz et al., 2012). Here, the simultaneous recording of responses transmitted by hundreds of axons reveals that some but not all fibers are sensitized under inflammation.

In conclusion, this study demonstrates that local rBoNT/A1 application ameliorates mechanical hypersensitivity in mice with local inflammation. Though, assessment of molecular mechanisms with *in vivo* calcium imaging of DRG neurons connected to the peripheral nerve endings of the hind paw revealed that the signal transmission from the periphery towards the soma remained unchanged. In the spinal cord, cleaved SNAP25 is clearly present in rBoNT/A1-treated animals. The data suggest that the efficacy of BoNT for the treatment of inflammatory pain possibly mediated *via* a more central mode of action.

## Data Availability Statement

The raw data supporting the conclusions of this article will be made available by the authors, without undue reservation.

## Ethics Statement

The animal study was reviewed and approved by Ethics Committees of the Royal Veterinary College, London and King’s College London.

## Author Contributions

The study was designed by BO, CP, MK, and SM. BO, VM, SL, and AF performed the experiments. The manuscript was prepared and written by BO, SL, VM, and MK. Except SM, who sadly passed away, all authors reviewed and approved the manuscript.

## Conflict of Interest

Authors CP, MK, VM, and SL were employed by Ipsen. AF was employed by Transpharmation. The remaining authors declare that the research was conducted in the absence of any commercial or financial relationships that could be construed as a potential conflict of interest. The authors declare that this study received funding from Ipsen. The funder had the following involvement with the study: provided the recombinant botulinum neurotoxin, suggested the dose being used and approved the manuscript.

## Publisher’s Note

All claims expressed in this article are solely those of the authors and do not necessarily represent those of their affiliated organizations, or those of the publisher, the editors and the reviewers. Any product that may be evaluated in this article, or claim that may be made by its manufacturer, is not guaranteed or endorsed by the publisher.

## Footnotes

^1^ invivostat.co.uk

## Supplementary Material

Supplementary Figure 1 Immunohistochemical analysis of SNAP25 and cleaved-SNAP25 in the footpad. **(A–E)** Representative images of SNAP25 positive nerve endings identified in various skin structures and at the neuromuscular junction (NMJ) in footpad muscles in control animals. **(F–J)** Representative images of cleaved SNAP25 only detected at the NMJ **(F)** in CFA/rBoNT/A1-treated animals. **(K–O)** An isotype control antibody confirmed the specificity of the staining in CFA/rBoNT/A1-treated animals. Bars: 20 μm. *N* = 5/group (CFA/vehicle, CFA/rBoNT/A1) and *N* = 3 (absolute control). **(P)** Quantification of cleaved SNAP25 in the injected hind paw indicated by a cumulative score of cleaved SNAP25 in the neuromuscular junctions of the skin in mice treated with CFA and either rBoNT/A or vehicle (gelatine phosphate buffer; GPB; *N* = 5/group) and in absolute control animals (*N* = 3). Mean ± SEM. ^****^*p* < 0.0001 CFA/rBoNT/A1 vs. CFA/vehicle; Mann-Whitney test.

Supplementary Figure 2 Histopathological evaluation rBoNT/A1 on CFA-induced inflammation in the footpad. **(A–E)** Representative images of Hematoxylin-Eosin (H&E) staining of the footpad taken from CFA-injected mice. **(A)** Inflammatory lesions at low magnification (dashed line). **(B)** Inflammatory lesions are composed of macrophages and mixed neutrophil infiltrates (arrow) with empty lipidic vacuoles (^*^). **(C)** Some areas present foamy macrophage infiltrates containing droplets of CFA (arrowheads). **(D,E)** H&E staining of the footpad collected from untreated (absolute control) animals. **(F)** Overall inflammatory process grading and **(G)** neutrophil infiltrate grading in CFA/rBoNT/A1, CFA/vehicle, and absolute control animals. Bars: (**A,D**; 200 μm; **B,C,E**; 20 μm). **(A–C)** CFA/vehicle treated animals, *N* = 5/group (CFA/vehicle, CFA/rBoNT/A1) and *N* = 3 (absolute control). Mean ± SEM. Mann-Whitney test.

## Abbreviations

AAV9: adeno-associated virus, serotype 9
BoNT: Botulinum neurotoxin
c-SNAP25: cleaved synaptosomal-associated protein 25
rBoNT/A1: recombinant botulinum neurotoxin, subtype A1
CFA: complete Freund’s adjuvant
DRGs: dorsal root ganglion neurons
GCaMP6s: ultrasensitive genetically encoded calcium indicator
GFP-Calmodulin-Myosin light chain kinase M13: subtype 6s
GPB: gelatine-phosphate buffer
SNAP25: synaptosomal-associated protein 25.
