# Supplementary figures and images for: Evaluation of Recombinant Botulinum Neurotoxin Type A1 Efficacy in Peripheral Inflammatory Pain in Mice
Source: Front Mol Neurosci. 2022 May 26;15:909835. doi: 10.3389/fnmol.2022.909835 (PMC9179158; doi:10.3389/fnmol.2022.909835)

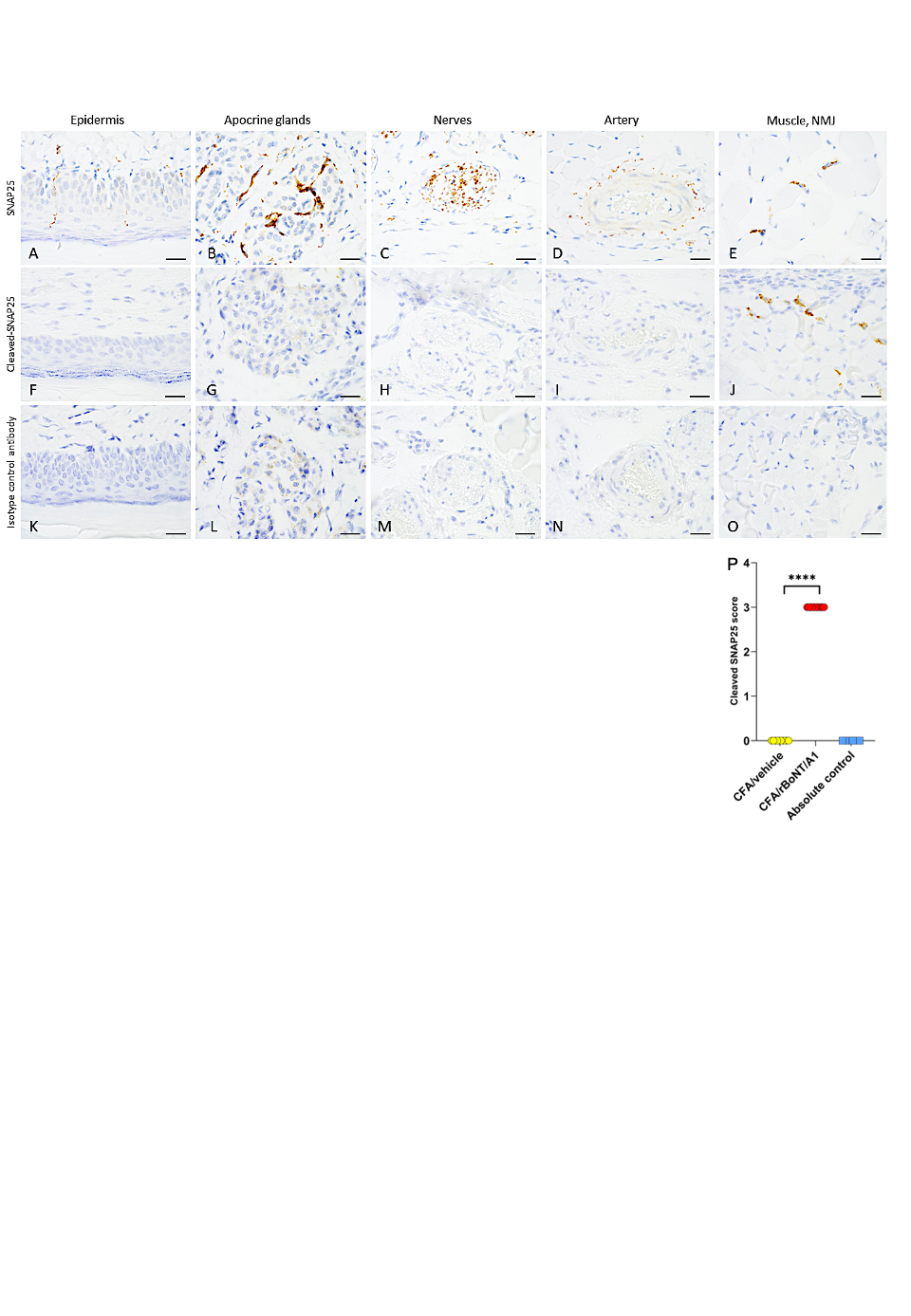

Supplement: Supplementary Figure 1 — Immunohistochemical analysis of SNAP25 and cleaved-SNAP25 in the footpad. (A–E) Representative images of SNAP25 positive nerve endings identified in various skin structures and at the neuromuscular junction (NMJ) in footpad muscles in control animals. (F–J) Representative images of cleaved SNAP25 only detected at the NMJ (F) in CFA/rBoNT/A1-treated animals. (K–O) An isotype control antibody confirmed the specificity of the staining in CFA/rBoNT/A1-treated animals. Bars: 20 μm. N = 5/group (CFA/vehicle, CFA/rBoNT/A1) and N = 3 (absolute control). (P) Quantification of cleaved SNAP25 in the injected hind paw indicated by a cumulative score of cleaved SNAP25 in the neuromuscular junctions of the skin in mice treated with CFA and either rBoNT/A or vehicle (gelatine phosphate buffer; GPB; N = 5/group) and in absolute control animals (N = 3). Mean ± SEM. ****p < 0.0001 CFA/rBoNT/A1 vs. CFA/vehicle; Mann-Whitney test. [file Image_1.TIFF]

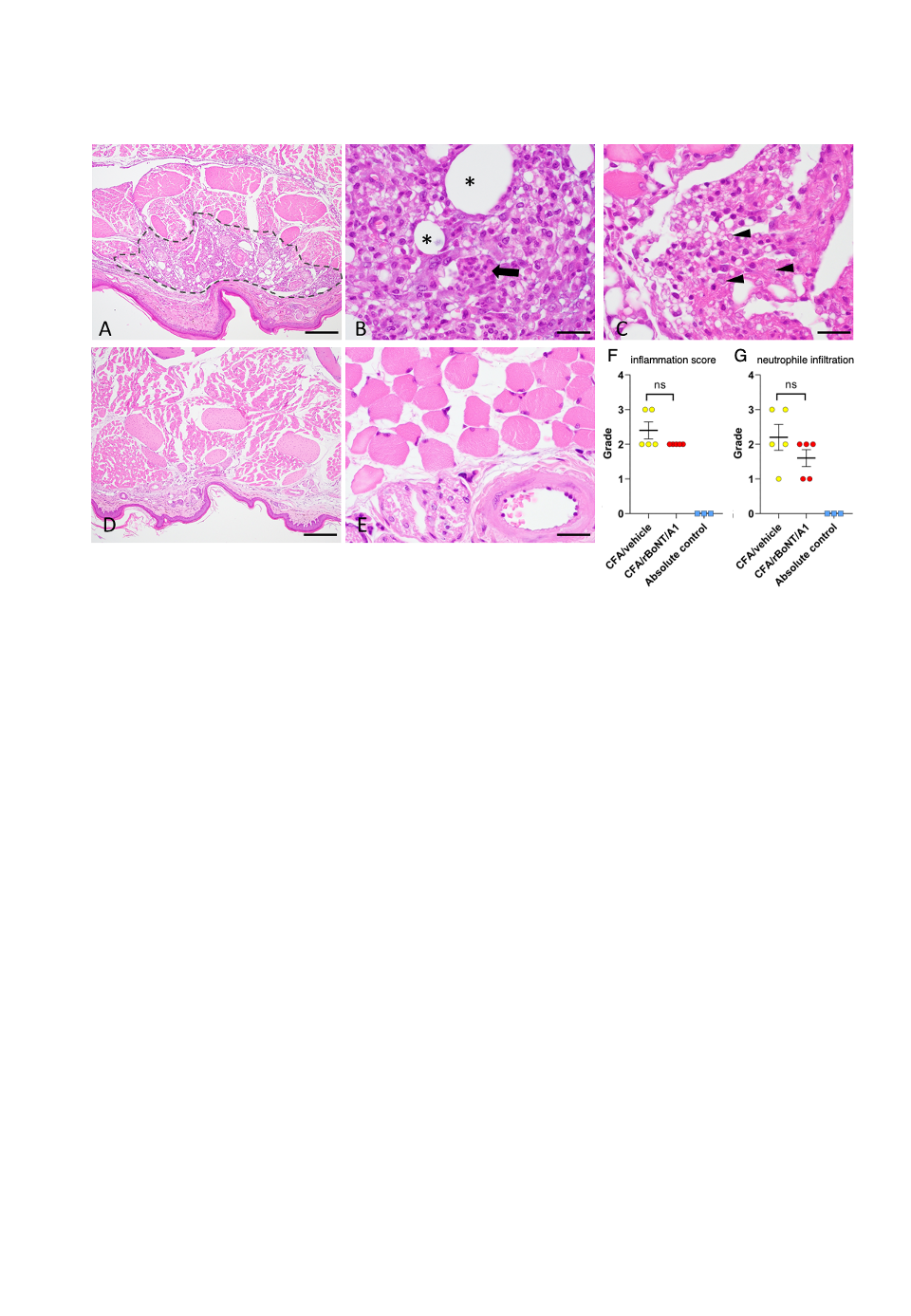

Supplement: Supplementary Figure 2 — Histopathological evaluation rBoNT/A1 on CFA-induced inflammation in the footpad. (A–E) Representative images of Hematoxylin-Eosin (H&E) staining of the footpad taken from CFA-injected mice. (A) Inflammatory lesions at low magnification (dashed line). (B) Inflammatory lesions are composed of macrophages and mixed neutrophil infiltrates (arrow) with empty lipidic vacuoles (*). (C) Some areas present foamy macrophage infiltrates containing droplets of CFA (arrowheads). (D,E) H&E staining of the footpad collected from untreated (absolute control) animals. (F) Overall inflammatory process grading and (G) neutrophil infiltrate grading in CFA/rBoNT/A1, CFA/vehicle, and absolute control animals. Bars: (A,D; 200 μm; B,C,E; 20 μm). (A–C) CFA/vehicle treated animals, N = 5/group (CFA/vehicle, CFA/rBoNT/A1) and N = 3 (absolute control). Mean ± SEM. Mann-Whitney test. [file Image_2.TIFF]
